# Characterization of MicroRNA Cargo of Extracellular Vesicles Isolated From the Plasma of *Schistosoma japonicum*-Infected Mice

**DOI:** 10.3389/fcimb.2022.803242

**Published:** 2022-02-28

**Authors:** Shun Li, Bikash R. Giri, Jingyi Liu, Xiaobing He, Pengfei Cai, Zhizhong Jing, Guofeng Cheng

**Affiliations:** ^1^ Shanghai Veterinary Research Institute, Key Laboratory of Animal Parasitology of Ministry of Agriculture and Rural Affairs, Chinese Academy of Agricultural Sciences, Shanghai, China; ^2^ Shanghai Tenth People’s Hospital, Institute for Infectious Diseases and Vaccine Development, Tongji University School of Medicine, Shanghai, China; ^3^ State Key Laboratory of Veterinary Etiological Biology, Lanzhou Veterinary Research Institute, Chinese Academy of Agricultural Sciences, Lanzhou, China; ^4^ Molecular Parasitology Laboratory, Queensland Institute of Medical Research (QIMR) Berghofer Medical Research Institute, Brisbane, QLD, Australia

**Keywords:** *Schistosoma japonicum*, schistosomiasis, extracellular vesicle, microRNA, plasma, lung stage, liver stage

## Abstract

*Schistosoma* is a genus of parasitic trematodes that undergoes complex migration in final hosts, finally developing into adult worms, which are responsible for egg production and disease dissemination. Recent studies documented the importance of extracellular vesicles (EVs) in the regulation of host-parasite interactions. Herein, we investigated the microRNA (miRNA) profiles of EVs isolated from host plasma at different stages of *Schistosoma japonicum* infection (lung stage: 3 days post-infection (dpi), and liver stages: 14 and 21 dpi) to identify miRNA cargo potentially involved in the pathogenesis and immune regulation of schistosomiasis. Characterization of the isolated plasma EVs revealed their diameter to be approximately 100 nm, containing typical EV markers such as Hsp70 and Tsg101. Deep sequencing analysis indicated the presence of 811 known and 15 novel miRNAs with an increasing number of differential miRNAs from the lung stage (27 miRNAs) to the liver stages (58 and 96 miRNAs at 14 and 21 dpi, respectively) in the plasma EVs of infected mice compared to EVs isolated from the uninfected control. In total, 324 plasma EV miRNAs were shown to be co-detected among different stages of infection and the validation of selected miRNAs showed trends of abundance similar to deep sequencing analysis. For example, *miR-1a-3p* and *miR-122-5p* showed higher abundance, whereas *miR-150-3p* and *miR-126a* showed lower abundance in the plasma EVs of infected mice at 3, 14, and 21 dpi as compared to those of uninfected mice. In addition, bioinformatic analysis combined with PCR validation of the miRNA targets, particularly those associated with the immune system and parasitic infectious disease, indicated a significant increase in the expression of *Gbp7*and *Ccr5* in contrast to the decreased expression of *Fermt3, Akt1*, and *IL-12a*. Our results suggested that the abundance of miRNA cargo of the host plasma EVs was related to the stages of *Schistosoma japonicum* infection. Further studies on the roles of these miRNAs may reveal the regulatory mechanism of the host-parasite interaction. Moreover, the differentially abundant miRNA cargo in host EVs associated with *S. japonicum* infection may also provide valuable clues for identifying novel biomarkers for schistosomiasis diagnosis.

## Introduction

Schistosomiasis, caused by parasitic flatworms of the genus *Schistosoma*, affects more than 230 million people in 78 tropical and subtropical countries (Weerakoon et al., 2015; McManus et al., 2020). It reportedly contributes to ~200,000 deaths annually and is considered a major health problem in Africa, the Middle East, Southeast Asia and South America (Colley et al., 2014). Schistosomes have a complex life cycle that require snails and mammalian hosts. After being released from snails, cercariae swim, and upon contact, penetrate the skin of the definitive host, and then transform into schistosomula. This transformation is associated with significant biochemical and physiological changes linked with the migration among several organs in the definitive host (Gobert et al., 2010). Firstly, schistosomula travel through the blood or lymphatic vessels of the pulmonary circulation to reach the lungs of final hosts. In mouse models of *Schistosoma mansoni* infection, schistosomula arrive at the lungs as early as 2 or 3 days of post-infection (dpi) and peak at 7 dpi (Miller and Wilson, 1978; Wheater and Wilson, 1979). However, *S. japonicum* could be recovered from the lung tissue at 2 dpi, whereas the number of these worms peak at 3 dpi (Sadun et al., 1958; Gui et al., 1995). Next, schistosomula can reach the liver either directly *via* the hepatic portal artery or by passing through the stomach, small intestine, spleen, or pancreas to the hepatic portal vein (Nation et al., 2020).

Intercellular communication is essential for multicellular organisms and is mediated *via* the transfer of secreted vesicles known as extracellular vesicles (EVs) (Thery et al., 2009; Liu et al., 2019). EVs are secreted by almost all types of cells, and contents of EVs include proteins, lipids, polysaccharides, and other molecules such as nucleic acids (O'Brien et al., 2020). Schistosome EV microRNAs (miRNAs) cargo had been shown to modulate the host immune response (Liu et al., 2019; Bischofsberger et al., 2020; Meningher et al., 2020). Moreover, the contents of the EVs released within an infected host can serve as promising biomarkers for the disease diagnosis and help in understanding the infection-induced host pathology (Meningher et al., 2017; Cai et al., 2020). Investigation of EVs isolated from sera of *S. mansoni*-infected individuals indicated that *bantam* and *miR-2c-3p* have promising potential for detecting the parasite infection, with a sensitivity of 80–86% and specificity of 84–94% (Meningher et al., 2017). Furthermore, exosomal miRNAs (*miR-92a-3p*, *miR-146a-5p*, and *miR-532-5p*) from human serum have been shown to distinguish patients with grade I–III fibrosis from patients without fibrosis, whereas only *miR-146a-5p* can differentiate subjects with mild (grade 0–I) and severe fibrosis (grade II–III) during *S. japonicum* infection (Cai et al., 2020).

In this study, we profiled miRNA cargo of EVs isolated from plasma of uninfected mice and infected mice (3 dpi for the lung stage; 14 and 21 dpi for the liver stage) at different stages. Next, several differentially abundant miRNAs were selected for RT-qPCR verification and several miRNA targets expression were analysed by RT-qPCR during *S. japonicum* infection.

## Materials and Methods

### Animals, *S. japonicum* Culture, and EV Isolation From Plasma

Male C57BL/6J mice (6–8 weeks old) were purchased from Shanghai SLAC Laboratory of Animal Co., Ltd, Shanghai (P.R. China). Animal experiments were carried out according to the recommendations in the Guide for the Care and Use of Laboratory Animals from the Ministry of Science and Technology of the People’s Republic of China. All animal procedures were approved by the Institutional Animal Care and Use Committee of Shanghai Veterinary Research Institute, Chinese Academy of Agriculture Sciences, P. R. China (Permit No. SHVRI-SZ-20200622-03).

The *S. japonicum* cercariae were provided from National Institute of Parasitic Diseases, Chinese Center for Disease Control and Prevention. Each mouse was challenged with 50 ± 2 *S. japonicum* cercariae *via* abdominal skin penetration. Five mice were randomly selected that were considered as a biological replicate. A total of 3 biological replicates were used for each experiment (n = 15/group). At 3, 14 and 21 dpi, blood samples were collected into K2 EDTA tubes, and then transferred into fresh tubes. The samples were centrifuged at 2000 × *g* for 30 min at 4°C. The supernatant was collected, transferred into a new tube, and then centrifuged at 12000 × *g* for 30 min. The EVs were isolated using the Exosupur Kit (Echo9101A, ECHO Biotech Co. Ltd., Beijing, P. R. China) and combined with ultracentrifugation. Briefly, plasma samples were diluted with phosphate buffered saline (PBS), then passed through 0.22 µm membrane filters and loaded onto the column. After washing the column, the EV-containing fractions were collected and pooled, followed by ultracentrifugation at 100,000 × *g* for 90 min at 4°C in a Beckman Coulter’s ultracentrifuge (Optima XPN-100, Beckman Coulter Inc., CA, USA). After removing the supernatant, the plasma EV pellet was resuspended in sterile PBS, and centrifuged at 100,000 × g for 90 min at 4°C. The supernatant was carefully removed, and the plasma EV was resuspended in an appropriate volume of PBS.

### Electron Microscopy and Nanosight Analysis

EV suspensions were adsorbed onto 200 mesh formvar-coated grids (Agar Scientific, Essex, UK) for 2 min at room temperature (25°C). The grids were then stained with 2% phosphotungstic acid for 2 min and examined under a transmission electron microscope (JEM1400, JEOL Ltd., Tokyo, Japan). The size distributions of EVs were determined by nanoparticle tracking analysis (NTA) using the NanoSight system (NanoSight, Malvern, UK).

### Western Blot

Mouse peripheral blood mononuclear cells (PBMCs) were isolated from whole blood by density centrifugation with 1.084 ficoll plaque. Plasma EVs and PBMCs from the hosts were homogenized in RIPA lysis buffer (Beyotime Biotechnology., Beijing, P. R. China) containing protease and phosphatase inhibitors (Thermo Fisher Scientific Corp., MA, USA). Proteins in the lysates were separated by sodium dodecyl sulphate 12% polyacrylamide gel electrophoresis and then transferred to polyvinylidene difluoride (PVDF) membranes. The membranes were blocked with 5% skim milk dissolved in Tris-buffered saline supplemented 0.1% of Tween 20 (TBST) for 1 h at room temperature and then incubated with anti-TSG101, anti-Hsp70, anti-Calnexin (1:1000 dilution, Abcam, Cambridge, UK) overnight at 4°C. Each membrane was washed in TBST thrice for 10 min each time and incubated with a horseradish peroxidase–conjugated goat anti-rabbit IgG secondary antibody (Thermo Fisher Scientific Corp, 1:5000 dilution), labeled with chemiluminescent HRP substrates (Merck, Darmstadt, Germany) and visualized with a ChemiDoc™ Touch Imaging System (Bio-Rad Laboratories). Images of the blot were acquired with Image Lab Software version 5.2.1 (Bio-Rad).

### Small-RNA Sequencing and Data Analyses

Total RNA was isolated from the EVs and subjected to library preparation. The RNA library was prepared from the 18–30 nt fraction extracted from a denaturing 15% polyacrylamide gel using the TruSeq Small RNA Library Preparation Kit (Illumina, CA, USA). The small-RNA libraries were subjected to Illumina 50 bp single end sequencing on the Illumina HiSeq 2500 platform. Raw reads in fastq format were pre-processed by in-house Perl scripts. Briefly, the sequences were cleaned by removing adapter sequences, reads containing poly-N, low-quality reads, and oligonucleotides with length >32 or <18 nt. The Bowtie tool was used to align and compare the clean reads with sequences in the Silva, GtRNAdb, Rfam, and Repbase databases (Langmead et al., 2009). Unannotated reads containing miRNAs were obtained by filtering out ribosomal-RNA sequences as well as transfer-RNA, small-nuclear-RNA, small-nucleolar-RNA, and repeated sequences. After filtering, the remaining reads were used to detect known miRNAs by comparison with the known miRNAs from miRBase v.22. Randfold tools were used for novel miRNA secondary structure prediction. Target gene functions were predicted according to the following databases: NCBI nr (non-redundant protein sequences), Pfam (Protein family), KOG/COG (Clusters of Orthologous Groups of proteins), Swiss-Prot, KEGG (KEGG Ortholog database), and GO (Gene Ontology). Quantification of miRNA abundance was estimated as follows: 1) Small RNAs were mapped back onto the precursor sequence; 2) Read count for each miRNA was obtained from the mapping results; and 3) Reads with the same unique molecular identifier (UMI) were normalized to 1. Differential abundance analysis between two groups was performed using the edgeR R package (3.12.1) (Robinson et al., 2010; McCarthy et al., 2012). The resulting *P* values were adjusted by the Benjamini Hochberg approach for controlling the false discovery rate (Benjamini and Hochberg, 1995).

### Prediction of MiRNA Targets and miRNA-Target Network

RNAhybrid (Kruger and Rehmsmeier, 2006), miRanda (Miranda et al., 2006), and TargetScan (Agarwal et al., 2015) were used to predict the targets of miRNAs, and the targets consistently predicted by at least two algorithms were considered miRNA target genes. For mouse miRNA target identification and target network construction, we used online tool miRTargetLink 2.0 (https://www.ccb.uni-saarland.de/mirtargetlink2) (Kern et al., 2021). Two highly differentially abundant (*miR-122-5p* and *miR-126a-5p*) were selected for network analysis, we only included the strongly validated targets with a pathway to build and visualize the network.

### RNA Extraction and Reverse-Transcription Quantitative Polymerase Chain Reaction

Total RNAs were extracted from EVs and PBMCs using TRIzol according to the manufacturer’s instructions (Invitrogen, Thermo Fisher Scientific Corp.), respectively. The isolated RNA was reverse-transcribed with the miScript II RT Kit (QIAGEN, Hilden, Germany). The abundance of miRNAs was determined using the miScript SYBR Green PCR Kit (QIAGEN), and *cel-miR-39-3p* was used as a spike-in control (QIAGEN). To quantify target gene expression level, real-time quantitative PCR was performed *via* the following thermal-cycling programme: 95°C for 5 min, followed by 40 cycles of 95°C for 10 s, 57°C for 20 s, and 72°C for 36 s. For evaluating the expression of target genes, glyceraldehyde-3-phosphate dehydrogenase (*GAPDH*) was amplified as an internal control. The fold change was calculated by the 2^−ΔCT^ method (Livak and Schmittgen, 2001). All the primer sequences are provided in **Supplementary Tables 1** **,** **2**.

### Statistical Analysis

Data were analysed and plotted in GraphPad Prism version 8.0.0 and shown as the mean ± standard error mean. Significance of differences between different groups were analysed by the unpaired two-sample *t* test. *P* ≤ 0.05 was considered statistically significant.

## Results

### Characterization of Mouse Plasma EVs

**Figure 1:**
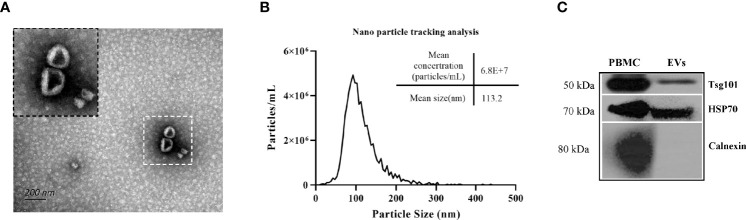
Characterization of mouse plasma derived EVs. **(A)** Electron microscopical analysis of EVs isolated from mouse plasma **(B)** Particle size distribution and concentrations of representative EVs were determined by nanoparticle tracking analysis of the EVs (isolated from uninfected-mouse plasma) **(C)** Immunoblots analysis of isolated EVs including typical EV markers such as Tsg101and Hsp70 and of non-exosomal marker Calnexin.

Transmission electron microscopy analysis of the isolated EVs showed a typical ‘cup shape’ (**Figure 1A**). The EV size analysis indicated that the particle diameter was ~100 nm (**Figure 1B**). Western blot analysis indicated that the EV proteins were able to recognized by anti-Hsp70 and anti-Tsg101 antibodies but not by anti-Calnexin antibody (**Figure 1C**). Because Hsp70 and Tsg101 are considered as representative markers of EVs, our results suggested that the isolated EVs were suitable for further analysis.

### MiRNA Cargo of EVs Isolated From the Plasma of Uninfected and *S. japonicum*-Infected Mice

**Figure 2:**
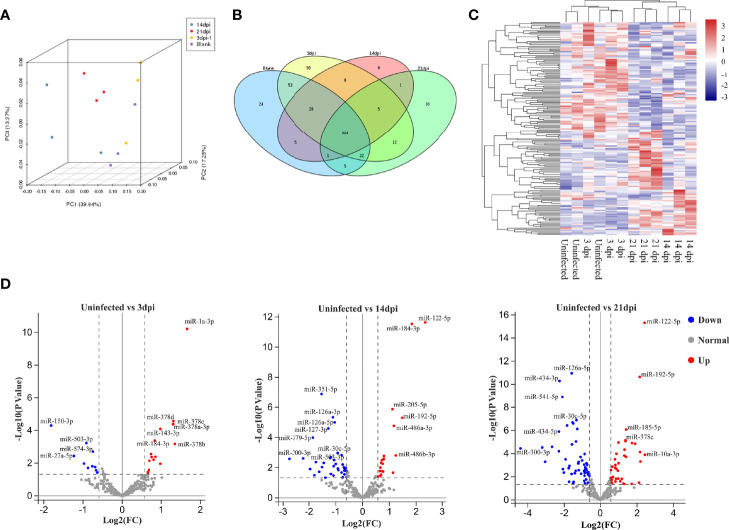
Comparative analysis of miRNA cargo from host plasma EVs during *S. japonicum* infection at different stages. **(A)** Principal component analysis plots for RNA seq data from each plasma EV. **(B)** Venn diagram of the identified miRNAs between plasma EVs isolated from different stages of *S. japonicum* infection and uninfected controls. **(C)** Hierarchical clustering analysis of differentially abundant miRNAs in plasma EVs from uninfected and *S. japonicum*-infected mice at different stages of infection. **(D)** Volcano plot of differentially abundant miRNAs in the EVs isolated from uninfected vs infected mice at 3, 14 or 21 dpi, respectively. Significantly higher abundance of EV-derived miRNAs is presented as red dots, whereas significantly lower abundance EV-derived miRNAs are indicated as blue dots. Several miRNAs with differentially highly increased or decreased abundance are marked in the plot, whereas normal miRNAs detected at all stages are marked with grey dots.

To assess variations among the different samples, all samples were subjected to principal component analysis. The results indicated that EV miRNAs isolated from the plasma of uninfected and infected mice at 3 dpi were closer, while difference was shown between the EVs at 14 and 21 dpi (**Figure 2A**). High-quality clean reads of 12 libraries, detailed read counts, GC%, and Q30% were listed after the removal of low-quality reads and adapter sequences (**Supplementary Data 1** **,** **2**). To identify EV miRNA cargo, small RNAs were analysed by filtering out ribosomal RNAs, small conditional RNAs, small nucleolar RNAs, small nuclear RNAs, and transfer RNAs. The remaining sequences were aligned to miRNA databases (miRBase v.22). In total, 826 miRNAs were identified in the 12 libraries containing 811 known and 15 novel miRNAs (**Supplementary Data 3**). MiRNA abundance heatmap showed that miRNAs from 3 dpi EVs were relatively similar to that from control EVs (**Supplementary Figure 1A**). Raw data were deposited in the CNGB Sequence Archive of China National GeneBank DataBase under the accession number CNP0002518 (Chen et al., 2020; Guo et al., 2020).

### Abundance of miRNAs in the EVs Isolated From Host Plasma at Different Stages during *S. japonicum* Infection

To determine the abundance of EV miRNA cargo during *S. japonicum* infection, we compared these data to the miRNA profile of EVs isolated from the plasma of uninfected mice. The results indicated that 324 miRNAs were shown to be co-detected among all the groups (**Figure 2B**). Subsequent hierarchical clustering of differentially abundant miRNAs yielded two clusters where uninfected and infected mice at 3 dpi shared a cluster, and those infected at 14 and 21 dpi shared another cluster (**Figure 2C**). Volcano plot analyses were also used to visualise the differentially abundant miRNAs in different *S. japonicum*-infected groups as compared to the uninfected control (**Figure 2D**). We retained only the miRNAs with average abundance (in transcripts per million) higher than 10 as the final abundant miRNAs to build the volcano plots. The results indicated a sharp increase in the number of differentially abundant miRNAs in host plasma-derived EVs from the lung stage (infected mice vs uninfected mice, 3 dpi: 27 miRNAs in total; 17 with higher and 10 lower abundance levels in infected mice) to the liver stages (infected vs uninfected mice, 14 dpi: 58 miRNAs in total; 21 with higher and 37 with lower abundance levels in infected mice; 21 dpi: 96 miRNAs in total; 39 with higher and 57 with lower abundance levels; log_2_[fold change] ≥ 0.584; *P* ≤ 0.05) (**Figure 2D**). Notable differences were also revealed between the following two comparisons: 1) infected mice at the 3 dpi lung stage vs infected mice at the 14 dpi liver stage (total: 68 co-detected miRNAs; 30 with higher and 38 with lower abundance levels at 14 dpi); 2) 3 dpi vs infected mice at the 21 dpi liver stage (total: 97 co-detected miRNAs; 35 with higher and 62 with lower abundance levels at 21 dpi). The differential abundance of miRNAs was also noted between the liver stages of 14 and 21 dpi (total: 53 co-detected miRNAs; 35 with higher and 18 with lower abundance levels at 21 dpi) (**Supplementary Figure 1B**). Based on the abundance of miRNAs at various stages, we performed K-means clustering analysis, which identified eight clusters with distinct abundance patterns. Cluster 1 (21 miRNAs) and cluster 8 (27 miRNAs) showed a pattern of increased abundance, whereas cluster 7 (27 miRNAs) manifested decreased abundance as compared to the uninfected control (**Supplementary Figure 2A**).

### RT-qPCR Verification of the Differentially Abundant miRNAs From the EVs Isolated from *S. japonicum*-Infected mice

**Figure 3:**
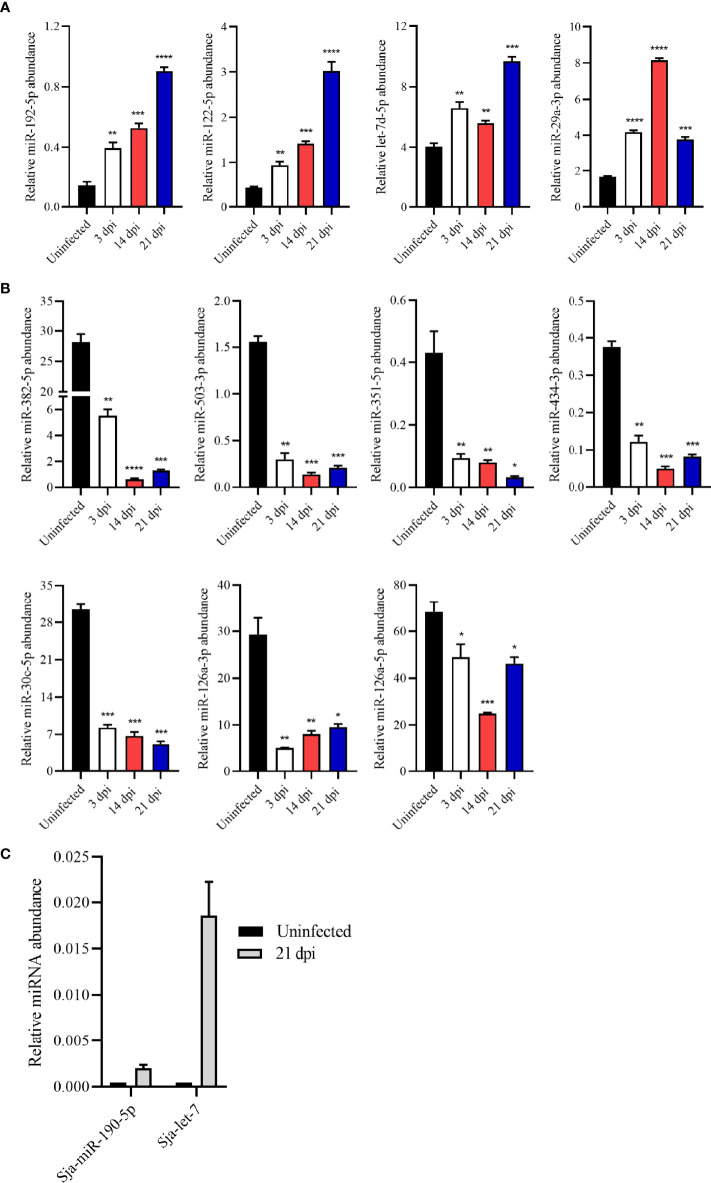
Validation of the abundance of miRNA cargo from host plasma EV by RT-qPCR. **(A)** Highly abundant miRNAs including *miR-192-5p*, *miR-122-5p*, *let-7d-5p* and *miR-29a-3p* showing significantly increased abundance in plasma EVs upon *S. japonicum* infection. **(B)** *MiR-382-5p*, *miR-503-3p*, *miR-351-5p*, *miR-434-3p*, *miR-30c-5p*, *miR-126a-3p* and *miR-126a-5p* showing decreased abundance in host plasma EVs upon *S. japonicum* infection. **(C)** *S. japonicum*-specific miRNAs showed relative abundance in plasma EVs isolated mice at 21 dpi as determined by RT-qPCR. *P < 0.05, **P < 0.01, ***P < 0.001, ****P < 0.0001 vs the uninfected group.

To verify the RNA-seq results, several miRNAs were selected and quantified their abundance in EVs by RT-qPCR. The results revealed higher abundance of miRNAs such as *miR-192-5p, miR-122-5p, let-7d-5p*, and *miR-29a-3p* in the plasma EVs from *S. japonicum*-infected mice than those from uninfected mice, which was consistent with the RNA-seq results (**Figure 3A**). Similarly, relative abundance of *miR-382-5p, miR-503-3p, miR-351-5p, miR-434-3p, miR-30c-5p*, *miR-126a-5p* and *miR-126a-3p* in plasma EVs from infected mice was found to be lower as compared to these from uninfected mice as determined by RT-qPCR (**Figure 3B**). Overall, the RT-qPCR results of these altered abundance of EV miRNAs were consistent with the RNA-seq results. Interestingly, the RNA-seq results also noted that several *S. japonicum* miRNAs including *Sja-miR-71a*, *Sja-miR-71b, Sja-miR-190-5p, Sja-let-7* and *Sja-miR-36a* were also detected in the plasma EVs of infected mice at 21dpi. RT-qPCR also confirmed the abundance of some of these *S. japonicum* miRNAs in the plasma EVs from infected mice at 21 dpi (**Figure 3C**).

### Prediction and Analysis of Targets of Differentially Abundant miRNAs at Different Stages of *S. japonicum* Infection

**Figure 4:**
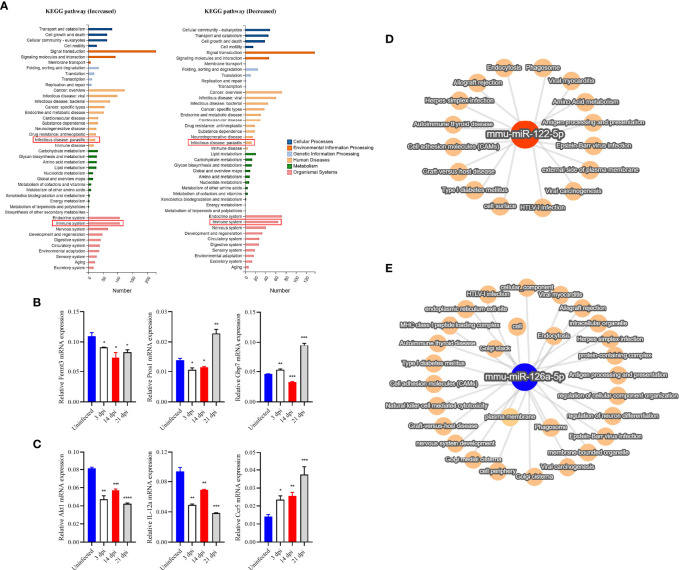
RT-qPCR verified the expression of selected target genes in PBMCs and KEGG and network analysis of selected target genes/miRNAs identified from host plasma EVs. **(A)** Target genes of differentially high- and low-abundance miRNAs as determined by KEGG pathway analysis. **(B)** The expression of selected target genes associated with the immune system were verified by RT-qPCR in PBMCs. The results showed decreased expression of *Fermt3* and partially increased expression of *Pros1* and *Gbp7*. **(C)** Likewise, target genes (of differentially high- and low-abundance miRNAs) associated with parasitosis were analyzed. The results indicated decreased expression of *Akt1*, and *IL-12a*, and increased expression of *Ccr5*. **(D, E)** The miRNA-mRNA target pathway network associated with other top differentially abundant miRNAs (*miR-122-5p* and *miR-126a-5p*) in EVs derived from the plasma of infected mice. The target pathway network showed their associations with several viral infections and immune response related functions such as antigen processing and presentation, also associated with plasma membrane, cell adhesion molecules, endocytosis and other terms. * *P* < 0.05, **P < 0.01, ***P < 0.001, ****P < 0.0001 vs the uninfected group.

Target prediction identified 8636 and 177 potential targets for 533 out of the 811 known miRNAs and for 10 out of the 15 novel miRNAs (**Supplementary Data 4**). We noted that there were 167 miRNAs being differentially abundant in the plasma EVs of infected mice as compared to those in uninfected mice (**Supplementary Data 5**). GO analysis of the target of altered abundance of miRNAs suggested that these miRNA targets are involved in cellular processes, single organism processes, biological regulation, and metabolic processes in biological processes (**Supplementary Figure 2B**) and are associated with growth, reproductive process, extracellular matrix, collagen trimer, nucleic acid binding transcription factor activity, transporter activity, receptor regulator activity, and guanyl-nucleotide exchange factor activity (**Supplementary Figure 2C**–**E**). In addition, we selected some differential miRNAs in the plasma EVs of infected mice at 3, 14 and 21 dpi as compared to those of uninfected mice (**Supplementary Data 6**) for KEGG pathway analysis which suggested these miRNA target genes are putatively involved in signal transduction, cancer (overview), endocrine system, infectious disease (viral, bacterial, parasitic) and immune system (**Figure 4A**).

### RT-qPCR Analysis of Expression of Selected Targets of Several miRNAs at Different Stages of *S. japonicum* Infection

To determine the potential regulatory role of EV miRNAs, we isolated PBMCs from *S. japonicum*-infected mice at 3, 14 and 21 dpi and then investigated the expression of several selected miRNA targets, which were predicted to be associated with the immune system and parasite-caused infectious diseases, by RT-qPCR (**Supplementary Data 7**). The results indicated that fermitin family member 3 (*Fermt3*, a target of *miR-122-5p*) was down-regulated, whereas protein S (*Pros1*, target of *miR-181b/d-5p*) and guanylate binding protein 7 (*Gbp7*, target of *miR-27a-3p*) was significantly up-regulated in the PBMCs isolated from infected mice at the late liver stage (21 dpi) as compared to those from uninfected mice (**Figure 4B**). Furthermore, thymoma viral proto-oncogene 1 (*Akt1*, a target of *miR-378a-3p*) and *IL-12a* (a target of *let-7d-5p*) were shown to be significantly decreased expression; chemokine (C-C motif) receptor 5 (*Ccr5*), which are targets of *miR-140-5p/3p* showed increased in PBMCs in infected mice as compared to those from uninfected controls (**Figure 4C**). Furthermore, the network analysis of two highly differentially abundant miRNAs (*miR-122-5p* and *miR-126a-5p*) indicated that these miRNAs are putatively associated with diverse functions: from infection to immune responses. For instance, *miR-122-5p* may be associated with external side of plasma membrane, cell surface, antigen processing and presentation, amino acid metabolism, virus infection, and other terms (**Figure 4D**). Similarly, differentially lower-abundance *miR-126a* was shown to be associated with different viral infections, endocytosis, cellular component, plasma membrane, and other terms (**Figure 4E**).

## Discussion

The discovery of miRNAs in EV cargo had demonstrated their important regulatory roles between cells and tissues. Additionally, these EV miRNAs are also considered to be a novel type of non-invasive biomarkers for diagnosing some diseases (Terlecki-Zaniewicz et al., 2019). To the best of our knowledge, no studies have characterized miRNA cargo in host plasma EVs at different stages of *S. japonicum* infection. Here, miRNA cargo in EVs isolated from the plasma of infected mice were characterized at different stages of *S. japonicum* infection including lung and liver stages.

Given that miRNAs play a critical role in the transmission of EV-mediated signals, we mainly focused on miRNA cargo in host plasma EV at different stages of *S. japonicum* infection. Specifically, we observed higher abundance of *let-7* and *miR-29* in plasma EVs from infected mice; these miRNAs are putatively involved in several key functions of the T-cell immune response. For instance, *let-7i* reportedly promotes the T helper 1 (Th1) immune response by aiding maturation of precursors to dendritic cells *via* inhibition of suppressor of cytokine signalling 1 and prevention of dendritic-cell-promoted expansion of regulatory T cells (Zhang et al., 2011). In contrast, *miR-29* can inhibit the Th1 immune response by directly targeting Eomes and T-bet, which are key transcriptional-regulator and effector molecules of Th1 responses, respectively (Steiner et al., 2011).

Our study also revealed several miRNAs with relative lower abundance in the EVs from the plasma of infected mice, for example *miR-126a, miR-382*, *miR-503*, *miR-351*, *miR-434*, and *miR-30c*. Among them, *miR-126a* has been shown to promote Th2 polarization in mice by targeting POU domain-class 2-transcription factor 3, and *miR-126a* inhibition leads to a reduced Th2 response (Mattes et al., 2009). Likewise, *miR-30* is associated with the development of other T cells (Podshivalova and Salomon, 2013). Furthermore, *miR-382-5p* supresses M1 macrophage activation and inflammatory responses by inhibiting CDK8 (Lv et al., 2021).

The expression of targets of several miRNAs was also analysed at different time points of infection (3, 14 and 21 dpi). We observed decreased expression of *IL-12a* in infected-mouse PBMCs as compared to the uninfected control. Since IL-12 production is regulated by P38 MAPK and hence is involved in the induction of the Th1 response (Romagnani, 2006). This results suggested that *S. japonicum* infection may affect the macrophage activity and associated immune response as reported previously (Xu et al., 2014). In addition, *IL-6* is essential for the Th1 immune response and is reported to participate in the up-regulation of TNF-α production and regulation of *Ccr5* expression. Remarkably, our data also suggested a significantly increased expression of *Ccr5* in PBMCs isolated from *S. japonicum*–infected mice, consistently with data on chronic helminth infections, which show a similar trend of expression characterized by a higher level of *Ccr5* and a lower level of *IL-12* as compared to control conditions (Rodriguez-Sosa et al., 2002). During the lung stage of infection, schistosomula remain in vasculature by suppressing immune activation of lung endothelial cells *via* targeting of integrins, *E-selectin*, and *Vcam1* (Trottein et al., 1999). High levels of TNF-α have been detected during *S. japonicum* infection and the concomitant increased expression of *Vcam1* (a *miR-126a-3p* target) supports the previous findings of their role in leukocyte recruitment during inflammation (Kong et al., 2018).

In the present study, our results revealed that *miR-122-5p*, *miR-184-3p*, and *miR-192-5p* have differentially higher abundance in plasma EVs from *S. japonicum*–infected mice at 14 and 21 dpi as compared to uninfected mice. Normally, *miR-122* is highly expressed in the liver, where it constitutes 70% of the total miRNA pool (Jopling, 2012), and plays a pivotal role in liver biology and disease (Bandiera et al., 2015). The result suggests that *S. japonicum* infection may be associated with significantly altered functions of host livers (Hu et al., 2020), whereas *S. haematobium* infection reduces the levels of cholesterol-rich lipoprotein species in overweight infected individuals and lower the risk of cardiometabolic diseases (Zinsou et al., 2020).

Additionally, *miR-122* has been suggested as a diagnostic marker of liver disease, but inconsistent levels of *miR-122* limit its potential as a diagnostic biomarker during *S. japonicum* infection (Cai et al., 2015). The differentially increased abundance of *miR-122* in plasma EVs at the liver stages, as observed in the present study, may be due to the *S. japonicum* infection–induced liver damage. The observed differentially higher abundance of *miR-122* in *S. japonicum*-infected host EVs is in agreement with a recent study that indicates its participation in hepatocyte innate immunity (Xu et al., 2019). Fermt3, also known as kindlin-3 (its mRNA is a target of *miR-122-5p*), binds and activates β-subunits of integrin and in turn results in the binding of integrin to many different ligands on a target molecule (Moser et al., 2008). Thus, both proteins participate in phagocytosis by promoting adhesion to opsonized particles (van Spriel et al., 2001). In the current study, we observed *Fermt3* down-regulation, which may be due to the regulation of a macrophage-mediated host immune response by worm-secreted EVs, as reported previously (Liu et al., 2019). *MiR-192-5p* is a conserved miRNA that is abundant in the liver and plays critical roles in several hepatic disorders, including chronic hepatitis B (Nielsen et al., 2018). Interestingly, increasing evidence suggests that *miR-192-5p* is closely related to various physiological and pathological processes (Ren et al., 2021). Moreover, exosomal miRNAs in serum have been proposed as potential markers for grading hepatic fibrosis in *S. japonicum* infected patients (Cai et al., 2020).

Interestingly, our results revealed the presence of five *S. japonicum*-specific miRNAs in the EVs isolated from host plasma at 21 dpi. Among them, *Sja-let-7* was highly enriched in all three biological replicates (average transcripts per million: 688888.86). Also, a number of studies have identified helminth-derived miRNAs in host biofluids, these RNAs may represent novel biomarkers and exert regulatory actions in a cross-species manner (Cheng et al., 2013; Hoy et al., 2014; Tritten et al., 2014; Hansen et al., 2016; Meningher et al., 2017; Lin et al., 2019; Liu et al., 2019). However, it remains to be investigated whether these schistosome-specific miRNAs can be actively taken up by host plasma EVs. Additionally, it is also possible that these *Schistosoma* specific miRNAs that secreted from parasite EVs released into host plasma, which were obtained when the host plasma EVs were collected. It is also necessary to take in consideration the possibility that the strain of animals selected for study may also affect the pathological outcomes and levels of circulating miRNAs.

In summary, our study indicated considerable differences in miRNA abundance profiles of host plasma EVs among different stages of *S. japonicum* infection. These differences in the abundance of infection stage-specific EV miRNAs indicate a potential regulatory role of several immune response related genes in PBMCs. Our findings improve the understanding of the host EV miRNA cargos isolated from plasma at lung and liver stages of *S. japonicum* infection. Further in-depth investigation on the functions of miRNAs reported in this study will significantly elucidate stage-specific schistosome-host interactions.

## Data Availability Statement

The datasets presented in this study can be found in online repositories. The names of the repository/repositories and accession number can be found below: CNGB Sequence Archive of China National GeneBank DataBase, CNP0002518.

## Ethics Statement

The animal study was reviewed and approved by the Animal Management Committee and the Animal care and Use committee of the Shanghai Science and Technology Commission of the Shanghai Municipal government for Shanghai Veterinary Research Institute, Chinese Academy of Agriculture Sciences, P. R. China (Permit No. SHVRI-SZ-20200622-03).

## Author Contributions

SL participated in performing experiments and formal analysis. BG participated in formal analysis, data visualization, and writing original draft. JL, XH, PC and ZJ edited and revised the manuscript. GC participated in conceptualization, editing manuscript, and supervision. All authors contributed to the article and approved the submitted version.

## Funding

This research was funded by the Research Fund for International Young Scientists (31950410564), from the National Natural Science Foundation of China (31472187 and 31672550) and the State Key Laboratory of Veterinary Etiological Biology, Lanzhou Veterinary Research Institute (SKLVEB2020KFKT018). The funders had no role in study design, data collection, analysis, decision to publish, or manuscript preparation.

## Conflict of Interest

The authors declare that the research was conducted in the absence of any commercial or financial relationships that could be construed as a potential conflict of interest.

## Publisher’s Note

All claims expressed in this article are solely those of the authors and do not necessarily represent those of their affiliated organizations, or those of the publisher, the editors and the reviewers. Any product that may be evaluated in this article, or claim that may be made by its manufacturer, is not guaranteed or endorsed by the publisher.
