# Supplementary material for: Characterization of MicroRNA Cargo of Extracellular Vesicles Isolated From the Plasma of Schistosoma japonicum-Infected Mice
Source: Front Cell Infect Microbiol. 2022 Feb 28;12:803242. doi: 10.3389/fcimb.2022.803242 (PMC8918519; doi:10.3389/fcimb.2022.803242)
Supplement: Supplementary file 8 [file Image_1.pdf]

A

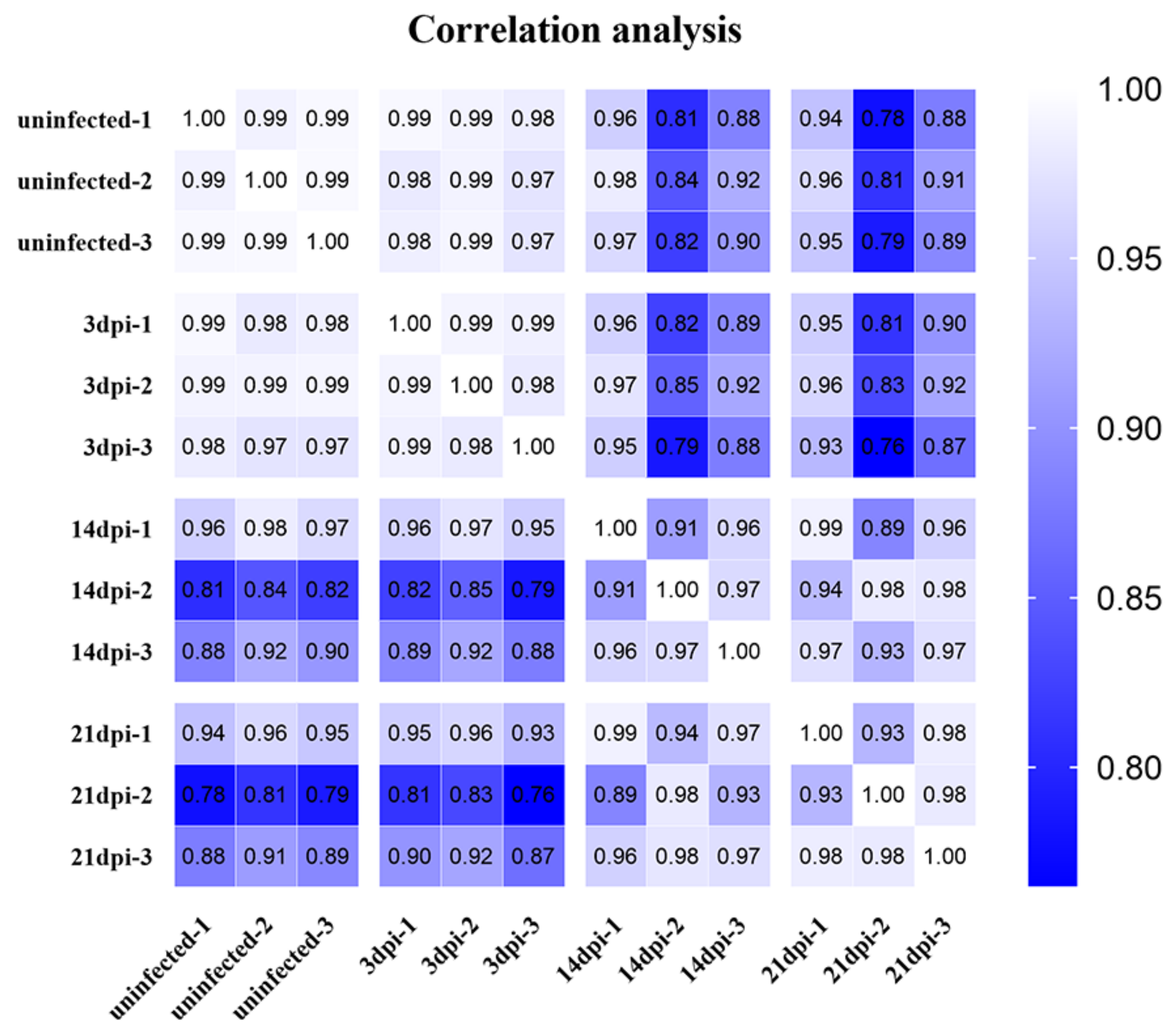


**Supplementary Figure 1. (A)** The Person's correlation heatmap of miRNAs abundance profiles;

B

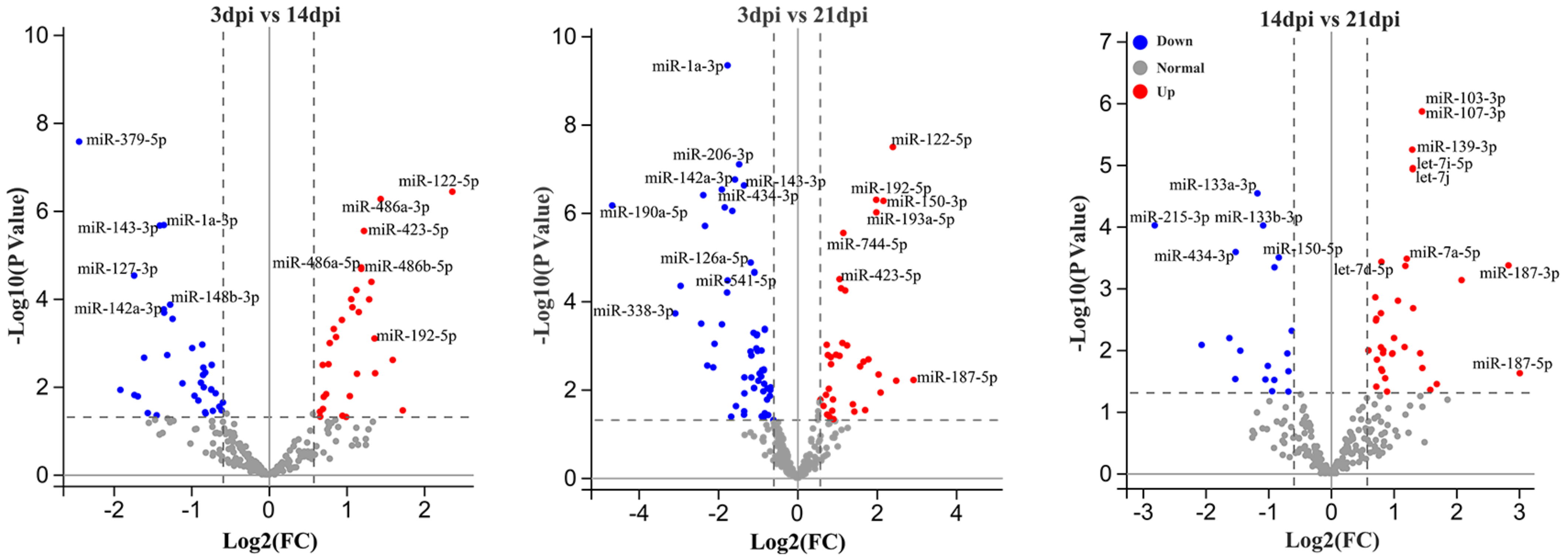


**Supplementary Figure 1. (B)** Volcano plot showing differentially abundant miRNAs within EVs isolated from lung stages 3 dpi vs liver stages 14 dpi and 21 dpi; and within liver stage 14 dpi vs 21 dpi. Significantly higher abundant miRNAs are shown as red dots, significantly lower abundant miRNAs marked as blue dots.
