# Supplementary material for: Characterization of MicroRNA Cargo of Extracellular Vesicles Isolated From the Plasma of Schistosoma japonicum-Infected Mice
Source: Front Cell Infect Microbiol. 2022 Feb 28;12:803242. doi: 10.3389/fcimb.2022.803242 (PMC8918519; doi:10.3389/fcimb.2022.803242)
Supplement: Supplementary file 9 [file Image_2.pdf]

A

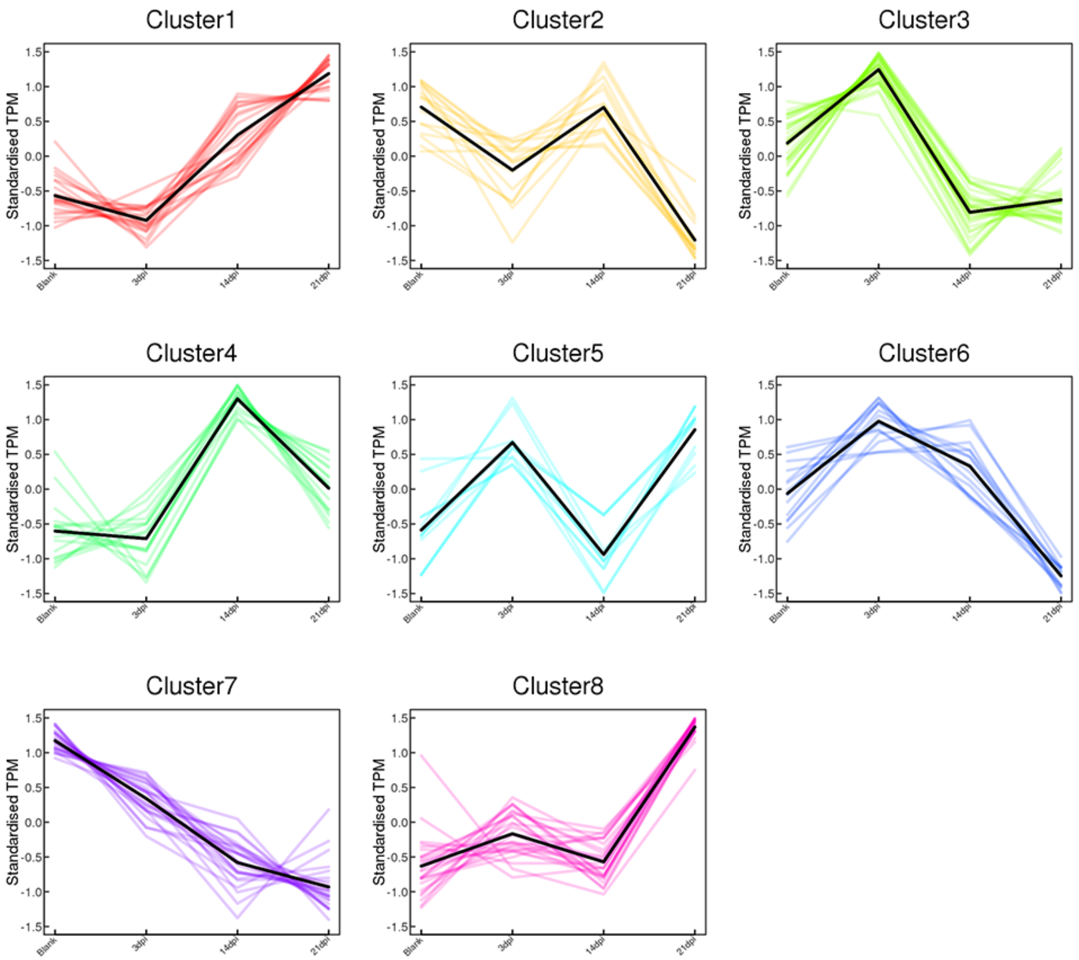

Supplementary Figure 2. (A) K-mean cluster analysis based on the abundance of miRNAs from isolated EVs at various stages of infection and uninfected mice.

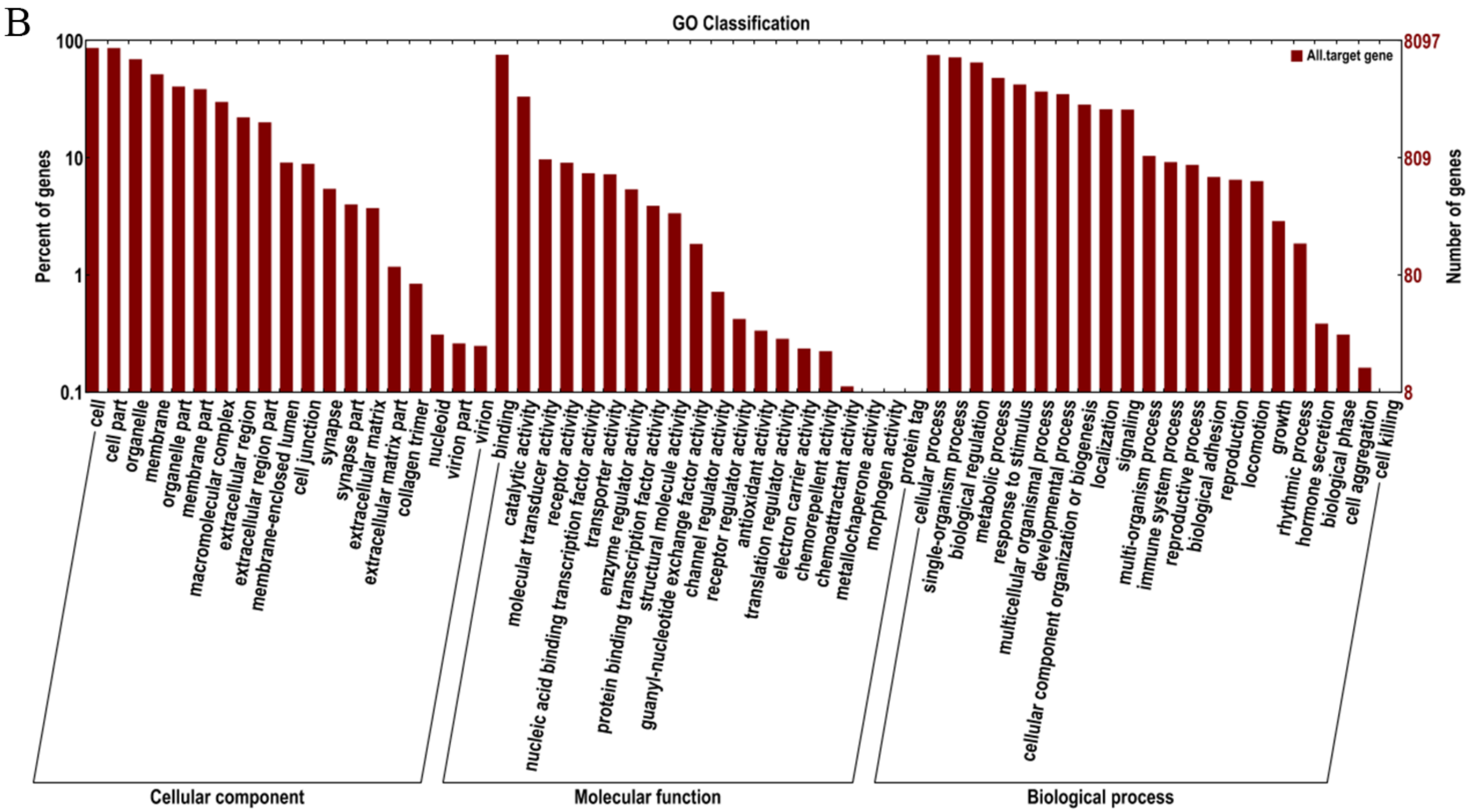

Supplementary Figure 2. (B) The GO term classification of all detected miRNAs target genes.

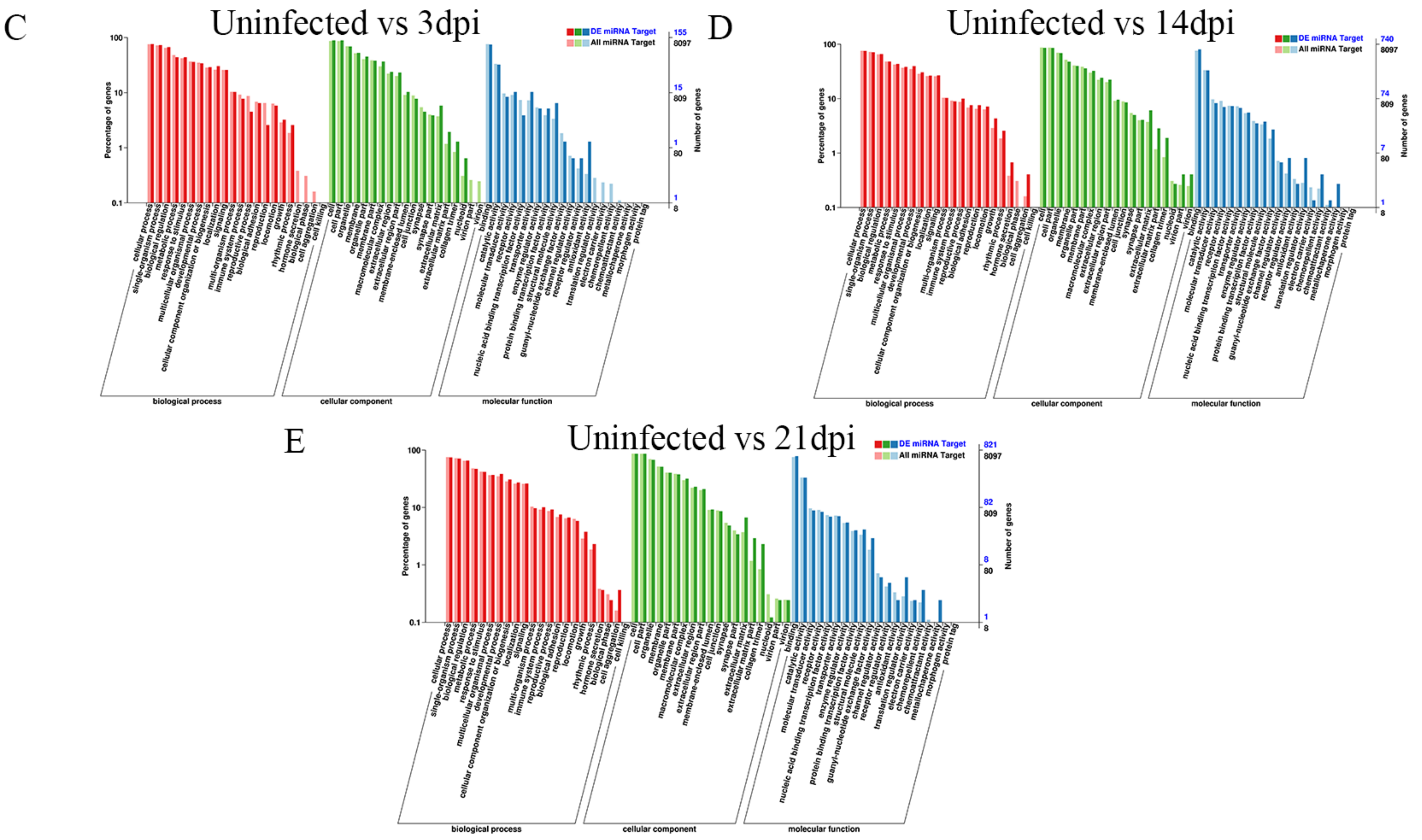

Supplementary Figure 2. (C) GO term classification of uninfected vs 3dpi; **(D)** uninfected vs 14dpi; **(E)** uninfected vs 21dpi.
