# Supplementary material for: Characterization of MicroRNA Cargo of Extracellular Vesicles Isolated From the Plasma of Schistosoma japonicum-Infected Mice
Source: Front Cell Infect Microbiol. 2022 Feb 28;12:803242. doi: 10.3389/fcimb.2022.803242 (PMC8918519; doi:10.3389/fcimb.2022.803242)
Supplement: Supplementary file 10 [file Table_1.docx]

**S. Table 1. List of RT-qPCR primers used for mouse EVs and *S. japonicum* miRNAs validation.**

| **miRNA** | **Primers (5'-3')** |
| --- | --- |
| Sja-miR-190-5p | TGATATGTATGGGTTACTTGGTG |
| Sja-let-7 | GGAGGTAGTTCGTTGTGTGGT |
| mmu-miR-126a-3p | TCGTACCGTGAGTAATAATGCG |
| mmu-miR-30c-5p | TGTAAACATCCTACACTCTCAGC |
| mmu-miR-351-5p | TCCCTGAGGAGCCCTTTGAGCCTG |
| mmu-miR-382-5p | GAAGTTGTTCGTGGTGGATTCG |
| mmu-miR-434-3p | TTTGAACCATCACTCGACTCCT |
| mmu-miR-503-3p | GAGTATTGTTTCCACTGCCTGG |
| mmu-miR-126a-5p | CATTATTACTTTTGGTACGCG |
| mmu-miR-192-5p | CTGACCTATGAATTGACAGCC |
| mmu-miR-122-5p | TGGAGTGTGACAATGGTGTTT |
| mmu-let-7d-5p | AGAGGTAGTAGGTTGCATAGTT |
| mmu-miR-29a-3p | TAGCACCATCTGAAATCGGTTA |
| cel-miR-39-3p | TCACCGGGTGTAAATCAGCTTG |
