# Supplementary material for: Characterization of MicroRNA Cargo of Extracellular Vesicles Isolated From the Plasma of Schistosoma japonicum-Infected Mice
Source: Front Cell Infect Microbiol. 2022 Feb 28;12:803242. doi: 10.3389/fcimb.2022.803242 (PMC8918519; doi:10.3389/fcimb.2022.803242)
Supplement: Supplementary file 11 [file Table_2.docx]

**S. Table 2. List of RT-qPCR primers used to evaluate miRNA target genes expression.**

| **Genes** | **Primers (5'-3')** |
| --- | --- |
| Fermt3 | Forward: ATGGCGGGTATGAAGACAGC  Reverse: CACCAATGTGCGACTCCCC |
| pros1 | Forward: CGCTTTCGGGTGCTACTGG  Reverse: CACTCTCGTTCAAGGTTGCC |
| Gbp7 | Forward: TCCTGTGTGCCTAGTGGAAAA  Reverse: CAAGCGGTTCATCAAGTAGGAT |
| Akt1 | Forward: ATGAACGACGTAGCCATTGTG  Reverse: TTGTAGCCAATAAAGGTGCCAT |
| IL12a | Forward: CTGTGCCTTGGTAGCATCTATG  Reverse: GCAGAGTCTCGCCATTATGATTC |
| Ccr5 | Forward: TTTTCAAGGGTCAGTTCCGAC  Reverse: GGAAGACCATCATGTTACCCAC |
| GAPDH | Forward: GGTGAAGGTCGGTGTGAACG  Reverse: ACCATGTAGTTGAGGTCAATGAAGG |
